# Determining a cost effective intervention response to HIV/AIDS in Peru

**DOI:** 10.1186/1471-2458-9-352

**Published:** 2009-09-18

**Authors:** Robert W Aldridge, David Iglesias, Carlos F Cáceres, J Jaime Miranda

**Affiliations:** 1 Department of Epidemiology & Public Health, University College London, London, UK; 2 Instituto de Medicina Tropical Alexander von Humboldt, Universidad Peruana Cayetano Heredia, Lima, Peru; 3 School of Public Health, Universidad Peruana Cayetano Heredia, Lima, Peru; 4 Institute of Studies in Health, Sexuality and Human Development, Lima, Peru; 5 Department of Epidemiology and Population Health, London School of Hygiene and Tropical Medicine, London, UK; 6 School of Medicine, Universidad Peruana Cayetano Heredia, Lima, Peru

## Abstract

**Background:**

The HIV epidemic in Peru is still regarded as concentrated - sentinel surveillance data shows greatest rates of infection in men who have sex with men, while much lower rates are found in female sex workers and still lower in the general population. Without an appropriate set of preventive interventions, continuing infections could present a challenge to the sustainability of the present programme of universal access to treatment. Determining how specific prevention and care strategies would impact on the health of Peruvians should be key in reshaping the national response.

**Methods:**

HIV/AIDS prevalence levels for risk groups with sufficient sentinel survey data were estimated. Unit costs were calculated for a series of interventions against HIV/AIDS which were subsequently inputted into a model to assess their ability to reduce infection transmission rates. Interventions included: mass media, voluntary counselling and testing; peer counselling for female sex workers; peer counselling for men who have sex with men; peer education of youth in-school; condom provision; STI treatment; prevention of mother to child transmission; and highly active antiretroviral therapy. Impact was assessed by the ability to reduce rates of transmission and quantified in terms of cost per DALY averted.

**Results:**

Results of the analysis show that in Peru, the highest levels of HIV prevalence are found in men who have sex with men. Cost effectiveness varied greatly between interventions ranging from peer education of female commercial sex workers at $US 55 up to $US 5,928 (per DALY averted) for prevention of mother to child transmission.

**Conclusion:**

The results of this work add evidence-based clarity as to which interventions warrant greatest consideration when planning an intervention response to HIV in Peru. Cost effectiveness analysis provides a necessary element of transparency when facing choices about priority setting, particularly when the country plans to amplify its response through new interventions partly funded by the GFATM.

## Background

Patterns of the human immunodeficiency virus (HIV) vary across countries and regions, and the most vulnerable populations are not necessarily equivalent internationally or across continents. Recent data from the Peruvian Ministry of Health states that as of 31 December 2007, 30,389 cases of HIV and 20,610 cases of acquired immunodeficiency syndrome (AIDS) had been reported in the general population since 1983[1,2]. The Peruvian HIV epidemic is predominantly concentrated in men who have sex with men (MSM), with lower rates found in female sex workers (FSW) and lower still in the general population[3,4], and virtually no cases are reported amongst injecting drug users since such practice is rare in Peru.

In 2001 the United Nations (UN) adopted a set of commitments aimed at the prevention and treatment of HIV/AIDS. Prior to implementation, analysis was performed to determine the necessary resources and likely cost implications that this would entail[5]. In 2002, Stover et al[6] attempted to determine the effect of expanding such preventative interventions on global transmission rates. Their findings suggested that a further 29 million new infections could be prevented worldwide by 2010[5].

Here we attempt to evaluate potential HIV prevention strategies in Peru in terms of their ability to reduce Disability Adjusted Life Years (DALYS) individually and in combination. Whilst the epidemic in this country seemed to have stabilized at least a decade ago, and antiretroviral treatment (ART) has been offered free of charge by the Ministry of Health since 2004, insufficient prevention efforts might increase the cost of treatment and affect its sustainability [1,7]. Moreover, a number of new prevention projects are currently being funded by the Global Fund for AIDS, Tuberculosis and Malaria (GFATM) in Peru and other countries with similar epidemics. New cost-effectiveness data from these projects can therefore be used together with monitoring and evaluation to, if necessary, revise planned interventions to ensure that new resources are used in the most effective manner.

## Methods

### Baseline prevalence levels of HIV/AIDS in Peru

HIV prevalence levels in Peru were estimated using the Estimation and Projection Package (EPP), following the methodology as used by the Joint United Nations Programme on HIV/AIDS and the World Health Organisation (WHO) in projecting the WHO/UNAIDS country estimates [8-10]. Sentinel surveillance data, as used in their country estimates [11,12], was incorporated in our calculations of prevalence. The three main groups with sufficient available sentinel data were: MSM, FSW and pregnant women - the size of these groups and length of time they are assumed to remain in these groups are presented in Table 1. Sentinel data was available for the period 1985 to 2002 and for the MSM, FSW and pregnant women groups there were a total of 21, 14 and 67 published sentinel survey values used in the analysis[11,12]. National data was then estimated by applying the concentrated epidemic curves for the individual risk groups countrywide. Baseline projections were calculated on the assumption that behavioural changes do not occur in the future. Full details of the methodology followed for this work have been published elsewhere[10,13,14].

**Table 1:** Population risk groups and their associated estimated population size used in EPP analysis

**Population**	**Population size (15 years +)**	**Details of estimation**
MSM	620,978*	Based on 2005 estimate [23]
FSW	25,500†	2002 figure (average) based national aids programme report to UNAIDS[49]
Pregnant women	587,000‡	2005 Figures based on births per year[50]
Remaining population	26,040,522	2005 Figures from UN Population data base and calculated by subtracting populations from [50]

*MSM remain in this group for lifetime

† FSWs are estimated to remain in this population for an average of 5 years and return to remaining population afterwards

‡ Pregnant women remain in population for one year and return to remaining population afterwards

### The GOALS model

The GOALS model has been used in previous global and regional HIV analyses to assess the ability of a variety of interventions against HIV/AIDS to reduce rates of transmission in these regions [6,13]. The GOALS model is a deterministic model that uses data in three main areas to project HIV prevalence and incidence: demography; sexual behaviour; and HIV and sexually transmitted infection (STI) rates. GOALS considers four different risk groups, the exact definition of which is specified by the user. For the purposes of this analysis the groups were defined as: MSM, FSW and their clients, men and women with multiple sexual partners, and men and women in steady sexual relationships.

Initial parameter ranges for behavioural and biological inputs were specified based on a review of the available literature (see Tables 2 and 3 for full details). Ten thousand simulations of the GOALS model were then computed using @RISK software[15] to perform iterations using the Monte Carlo simulation method. Each iteration of the model sampled parameter values randomly from the uniform distributions defined by the ranges detailed in Tables 2 and 3. For each iteration, projected HIV prevalence levels for the period 1999 to 2004 were compared to those calculated by the baseline EPP analysis described in the last section which predicted baseline prevalence levels for HIV in Peru based on sentinel survey data. The set of behavioural and biological inputs within the GOALS model that minimized the deviation of HIV prevalence from the projected EPP analysis across the period 1999 to 2004 was then identified as the best-fit set of values to be used within the subsequent calculations and analysis of intervention effects.

**Table 2:** Behavioural parameters used in the GOALS model: Ranges and best fit values.

Input	Min	Max	Best fit	Source
Percent of men 15-49 that are sexually active	88.0	95.3	94.0	[39,51]
Percent of women 15-49 that are sexually active	74.8	95.5	91.0	[39,51]
Sexually active 15-19 as % of 15-49	54.2	76.8	67.0	[51]
				
MSM				
Input	Min	Max	Best fit	Source
Percent of men in risk group	0.0	15.0	3.2	[23,39,46]
coital frequency (acts per year)	30	70	35.7	[13]
number of partners per year	1	6	1.2	[39]
Condom usage (percentage of acts)	24.3	55.6	53.6	[46]
				
High risk men				
Input	Min	Max	Best fit	Source
Percent of men in risk group	4.0	36.0	4.4	[39,51]
coital frequency (acts per year)	2	70	10.0	[13,47]
number of partners per year	2	89	2.5	[13]
Condom usage (percentage of acts)	59.6	95.8	93.9	[7]
				
Medium risk men				
Input	Min	Max	Best fit	Source
Percent of men in risk group category	5.0	16.0	12.4	[13,52]
coital frequency (acts per year)	30	70	31.0	[13]
number of partners per year	2	5	2.2	†
Condom usage (percentage of acts)	7.5	36.6	19.2	[51]
				
Low risk men				
Input	Min	Max	Best fit	Source
Percent of men in risk group category	*	*	*	*
coital frequency (acts per year)	30	70	38.9	[13]
number of partners per year	1	2	1.1	†
Condom usage (percentage of acts)	0.0	28.9	23.8	[51]
				
Partners per year Women (percentage of total female population aged 15-49 in risk group)				
Input	Min	Max	Best fit	Source
High risk (< 0.0%)	500	1200	559.5	[13,52]
Medium Risk (18.5%)	1	3	1.5	†
Low risk (81.5%)	1	2	1.0	†

*Determined as remainder of MSM, High and Medium risk groups

† Number of partners per year for the low and medium risk groups provides the basis of stratification by which the risk group is defined.

**Table 3:** Biological parameters used in the GOALS model: Ranges and best fit values.

MSM				
				
Input	Min	Max	Best fit	Source
HIV prevalence	0.0	21.97	7.5	[12,24,46]
Prevalence of ulcerative STIs*	0.0	72.3	23.7	[23]
Prevalence of non-ulcerative STIs†	0.0	6.3	5.2	[53]
Percent of STI cases treated	84.1	87.1	85.3	[51]
				
High risk men				
				
Input	Min	Max	Best fit	Source
HIV prevalence	0.7	13.8	7.3	[54]
Prevalence of ulcerative STIs*	0.02	29.9	10.4	[23,54]
Prevalence of non-ulcerative STIs†	0.7	6.2	5.8	[23]
Percent of STI cases treated	84.1	87.1	85.8	[51]
				
Medium risk men				
				
Input	Min	Max	Best fit	Source
HIV prevalence	0.1	0.7	0.6	[54]
Prevalence of ulcerative STIs*	1.0	15.2	6.8	[54]
Prevalence of non-ulcerative STIs†	0.0	3.0	2.6	Assumption
Percent of STI cases treated	84.1	87.1	84.3	[51]
Condom usage				
				
Low risk men				
				
Input	Min	Max	Best fit	Source
HIV prevalence	0.1	0.6	0.6	[23,54]
Prevalence of ulcerative STIs*	0.8	7	3.3	[53]
Prevalence of non-ulcerative STIs†	0.0	4.7	2.8	[39,53]
Percent of STI cases treated	84.1	87.1	86.0	[51]
				
Women				
				
Input	Min	Max	Best fit	Source
HIV prevalence - FSW	0.6	1.9	1.8	[12]
HIV prevalence - Medium	0.1	1.9	0.5	Assumption
HIV prevalence - Low	0.1	1.1	0.1	[12]

* For the purposes of this analysis, ulcerative STIs include: syphilis, chancroid and herpes simplex virus-2.

† For the purposes of this analysis, non-ulcerative STIs include: gonorrhea and chlamydia.

### Calculating the costs of interventions

Unit costs for prevention interventions (per person reached) were calculated using a methodology published jointly by Asian Development Bank, UNAIDS, Futures Group International and Ease International[16]. The outputs of this software were specifically designed to work with the GOALS model and have been implemented previously in Indonesia[16]. Unit costs applied in estimating final values based upon on either: published Peruvian data; field experience of local experts working at local NGOs the authors held discussions with; or finally from direct programme experience of the Peruvian-based authors. Assumptions about personnel-to-participant ratios presented in the supplementary material are based upon values used in prior analysis[16] and expert local opinion [see Additional file 1]. Detailed unit costs used to calculate the cost per person reached for each intervention are presented in the supplementary material accompanying this article [see Additional file 1].

### Modelling HIV Interventions effects in Peru

Interventions individually against HIV, and then in combination, were assessed against a "no intervention" scenario, with the outcome measures of cost per infection averted and DALYS [17] used to assess effectiveness. The "no intervention" scenario was calculated by setting the coverage of all other interventions other than those being assessed, including condom usage, to zero. A number of interventions are available in GOALS, those chosen for inclusion in this study were:

• Mass media - media campaigns targeted at the general population. Costing is based on an average campaign price with four promotions per year - newspaper inserts delivered to 7% of the population, 52 TV emissions and 180 radio transmissions - reaching half of the adult population.

• Voluntary counselling and testing - the number of tests required is equal to twice the number of people infected with HIV. It was assumed that people will request a new test every five years.

• Peer counselling and treatment of sexually transmitted infections for female sex workers- the need is assumed to include all sex workers. Peer educators are trained by health professionals and provide condoms during counselling sessions.

• Peer counselling and treatment of sexually transmitted infections for MSM - outreach programmes similar to peer counselling for sex workers - condoms are provided during counselling sessions.

• Youth - includes children in the formal education system aged between 10 and 18. Sessions occur during normal school activities and are run by teachers who have undergone specific training.

• Condoms - public sector condom distribution, male type only. Demand is calculated by sexually active population, proportion of sexually active population in high and medium risk groups, number of partners, and number of contacts per partner in each risk group. Condoms are included as part of other interventions including all peer education interventions and STI treatment.

• STI treatment - Available to anyone requesting this activity. Prevalence rates are specified for each risk group in Table 3. Activity includes costs of treatment, counselling and condom provision. Drugs costs based on an average for treatment of urethral and vaginal discharge, genital ulcers and pelvic inflammatory disease.

• Prevention of mother to child transmission - includes counselling pre and post test, and nevirapine for mother and child if accepted.

• Highly active antiretroviral therapy - based on the three alternative treatment regimes proposed in a report addressing the implementation of an antiretroviral programme for Peru [1]. The basic programme includes: ARVS, Monitoring (CD4 + Viral Load). Intermediate programme is comprised of: Basic programme activities and imaging tests, additional lab monitoring, administrative costs. The comprehensive programme consists of: Intermediate programme activities and treatment of opportunistic infections.

The interventions outlined above influence rates of HIV transmission in GOALS by altering behavioural factors including: rates of condom use; treatment of STIs; number of sexual partners and age at first sex. This impact data was determined by review of the literature initially reported in 2004[18] which has been periodically updated in the GOALS model since and the version used in this analysis was last updated in February 2006[19]. Full details of the assumed impact of each intervention on rates of condom use; treatment of STIs; number of sexual partners and age at first sex is supplied in the supplementary material for this article [see Additional file 1].

DALYS averted were calculated using the number of infections averted per year and translating this into a new figure of years of life lost (YLLs) due to premature death and equivalent 'healthy' years lost due to disability (YLDs). The full calculation of the DALYs uses the methodology described by Fox-Rushby et al[20]. Spectrum was used to project HIV incidence and deaths in 5 year intervals (ages 15-80+). Survivorship with HAART was calculated using recently published Peruvian data[21].

### Uncertainty Analysis

Many of the behavioural and prevalence values used in the GOALS model have a level of uncertainty associated with them and in order to analyse the effects these have on the results, two sets of uncertainty analysis were carried out. Firstly, the effectiveness of interventions was recalculated using the values of low, average and high intervention effectiveness as included within the GOALS model [see Additional file 1]. Additionally, 10 separate computations of the model were run using randomly chosen values for the behavioural and biological parameters that created HIV prevalence rates which did not deviate by greater than 50% from the baseline predictions. Only biological parameters listed in table 3 were included within this analysis, those in the supplementary materials [see Additional file 1 - Stable 9] accompanying this report were not. Finally, a separate calculation of baseline prevalence rates was carried out using a prior version of EPP[22] for comparison of results.

### Ethics

The study protocol was reviewed and approved by Universidad Peruana Cayetano Heredia's Independent Review Board.

## Results

### Estimated prevalence levels of HIV in Peru - baseline projection data

The three main groups for which there were sufficient data available to make estimates of prevalence were: female sex workers, MSM and pregnant women. For 2008, the total number of people living with HIV was estimated as 63,795, a prevalence of 0.32%. Prevalence levels for MSM, FSW and pregnant women were 8.93%, 2.1%, and 0.15% respectively.

### Costs per person reached for prevention and treatment of HIV/AIDs

Table 4 summarises the unit costs per person reached by the interventions for prevention and treatment activities. Estimates for prevention activities were based on target group sizes of 1000 people and therefore economies of scale were ignored. Personnel and peer educator costs formed the highest proportion of most prevention strategies and final values are therefore extremely sensitive to assumptions about these values. Unit costs varied from $US 8 per youth reached by peer educator, up to $US 124 per commercial sex worker reached. HAART costs varied from $US 3379 for a basic programme to $US 3792 for a comprehensive package.

**Table 4:** Unit costs per person reached by intervention in 2008 $US.

**STI treatment**	**$US**
Cost per STI case treated	3.7
	
**Youth**	**$US**
Cost per youth reached by peer educator	8.0
Cost per teacher trained	62.0
	
**Commercial sex worker**	**$US**
Cost per sex worker reached	124.0
	
**Mass media**	**$US**
Cost per person reached with mass media	0.4
	
**MSM**	**$US**
Cost per person reached	103.0
	
**Voluntary counselling and testing**	**$US**
Total costs per client	40.0
	
**Prevention of mother to child transmission**	**$US**
Cost per mother and child treated	23.2
	
Antiretroviral programmes*	**$US**
Basic†	3379
Intermediate‡	3788
Comprehensive§	3792

Further details of the individual unit costs and assumptions made for each intervention are provided in the supplementary materials that accompany the manuscript [see Additional file 1].

*Costing for highly active antiretroviral therapy (HAART) were computed based on local protocols and prior analysis[1].

† Antiretrovirals, Monitoring (CD4 + Viral Load)

‡ Basic programme + Imaging tests, additional lab monitoring, administrative costs

§ Intermediate programme + treatment of opportunistic infections

### Projections of the effects of interventions

Tables 2 and 3 detail what was calculated as the best fit set of biological and behavioural parameters for the baseline prevalence estimates in GOALS. Table 5 shows the results for the number of infections averted and the cost effectiveness of these interventions expressed in terms of cost per infection averted and cost per DALY averted. The results suggest that if cost effectiveness were the only consideration when choosing which interventions to implement first then peer education of female sex workers, blood safety, peer education of youth in school and voluntary counselling and testing would be ranked ahead of others. Blood safety, condom distribution, and peer education of both, youth in school and MSM, provided the greatest rates of total infections averted. However, it must be noted that these values of total infections averted should not be compared against current epidemiological estimates as they do not take into account existing interventional impacts on condom usage, treatment of STIs and reduction in high risk behaviour which were set to zero for the purposes of this part of the analysis.

**Table 5:** Interventions against HIV/AIDS and their associated coverage levels, total costs, yearly costs, yearly infections averted, yearly DALYS averted and sorted by average cost effectiveness

**Intervention**	**Coverage level (%)**	**Total costs ($ Millions) 2008-2013**	**Yearly costs ($ Millions)**	**Total infections averted (2008-2013)**	**Yearly infections averted**	**Yearly DALYS averted**	Average cost effectiveness - $/DALY averted (range*)
FSW	50	0.6	0.1	1,970	394	10,118	55 (2 - 124)
FSW	95	1.0	0.2	3,730	746	19,157	55 (1 - 105)
FSW	80	0.9	0.1	3,130	626	16,076	55 (2 - 116)
Blood safety	50	5.3	0.9	13,050	2,610	67,025	79 (41 - 79)
Blood safety	80	8.5	1.4	20,570	4,114	105,648	80 (43 - 80)
Blood safety	95	10.1	1.7	24,260	4,852	124,599	81 (43 - 91)
VCT	95	8.2	1.4	13,870	2,774	71,236	116 (18 - 116)
VCT	50	4.4	0.7	7,300	1,460	37,493	118 (18 - 118)
VCT	80	7.1	1.2	11,670	2,334	59,937	118 (18 - 118)
Youth: in-school	50	13.7	2.3	21,760	4,352	131,126	104 (12 - 177)
Youth: in-school	80	22.0	3.7	22,760	4,552	137,152	161 (19 - 270)
Youth: in-school	95	26.2	4.4	23,020	4,604	138,719	189 (12 - 630)
mass media	50	11.3	1.9	8,930	1,786	45,864	246 (89 - 253)
Condoms	95	61.0	10.2	23,740	4,748	121,929	500 (64 - 500)
Condoms	80	51.3	8.6	19,890	3,978	102,155	503 (62 - 503)
Condoms	50	32.1	5.3	12,430	2,486	63,840	503 (58 - 503)
STI treatment	50	25.1	4.2	6,600	1,320	33,898	739 (200 - 1021)
STI treatment	80	40.1	6.7	10,290	2,058	52,849	759 (199 - 1045)
STI treatment	95	47.6	7.9	12,080	2,416	62,043	767 (200 - 1055)
MSM	50	75.0	12.5	11,090	2,218	56,958	1,317 (354 - 1317)
MSM	80	120.0	20.0	17,570	3,514	90,240	1,330 (351 - 1330)
MSM	95	142.5	23.8	20,770	4,154	106,675	1,336 (350 - 1336)
HAART							
Comprehensive	95	37.7	6.3	-	-	18917	1995
Intermediate	95	37.7	6.3	-	-	16412	2297
Basic	95	33.6	5.6	-	-	13999	2403
PMTCT	80	246.4	41.1	10,460	2092	41,557	5,928 (2313 - 5.928)

*Ranges based upon sensitivity analysis whereby the model was run using randomly generated values for the behavioural and biological parameters which were varied so that HIV prevalence rates did not deviate by greater than 50% from the baseline predictions.

**Figure 1:**
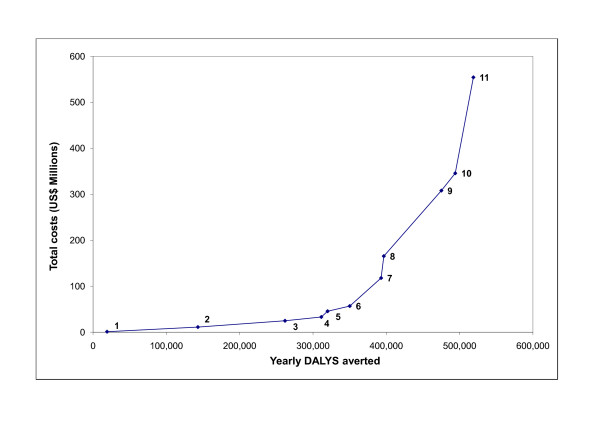
**Expansion pathway for incremental addition of interventions - based upon the average cost effectiveness ratio ($/DALY averted)**. 1= FSW 95% coverage; 2 = 1 + Blood safety 95% coverage; 3 = 2 + Youth: in-school 50% coverage; 4 = 3 + VCT 95% coverage; 5 = 4 + Youth: in-school 95% coverage; 6 = 5 + mass media; 7 = 6 + Condoms 95% coverage; 8 = 7 + STI treatment 95% coverage; 9 = 8 + MSM 95% coverage; 10 = 9 + HAART comprehensive; 11 = 10 + PMTCT 80% coverage.

Figure 1 shows the incremental benefits of each additional intervention as it is added to the package of interventions. The order in which interventions were added is based entirely upon the average cost effectiveness ratio ($/DALY averted) as detailed in Table 5.

### Sensitivity of the analysis to changes in intervention impacts

A series of sensitivity analyses were carried out to determine the impact of various assumptions within the model on the relative rankings of the cost effectiveness of each intervention. The effectiveness of interventions was recalculated using the values of low, average and high intervention effectiveness as included with the GOALS model. The effectiveness of interventions had impacts on the following factors: rates of condom use; treatment of STIs; number of sexual partners and age at first sex. Increasing the interventional effectiveness to a higher level had very little impact on the rankings apart from voluntary counselling and testing moved ahead of blood safety. Reducing the rankings to a lower level had more of an impact on the ranking of interventions: blood safety became the most cost effective, followed by peer education of youth and then voluntary counselling and testing.

In addition to this, 10 separate computations of the model were run using randomly chosen values for the behavioural and biological parameters that created HIV prevalence rates which did not deviate by greater than 50% from the baseline predictions. This analysis showed that for total infections averted over the five year period youth in-school, condoms and FSW showed the greatest absolute range of values, and blood safety, mass media and MSM interventions varied the least. In terms of rankings of the interventions based upon average cost effectiveness ratio ($/DALY averted) the model remained fairly stable. FSW and MSM both varied by only one place, the most variation across the rankings was shown by youth education (varying from first to fifth place) and condoms (varying from fourth to seventh place).

A separate calculation of baseline prevalence rates was carried out using a prior version of EPP[22] for comparison. Main differences in the modelling of data between the two versions were that the newer version of the software allows more accurate initial estimates of prevalence for individual risk groups and allows reassignment of populations between risks groups which particularly impacts the FSWs modelling in this work. Results of this analysis showed that for 2008, the total number of people living with HIV was estimated as 62,910; 43,799 of which were male, and 19,112 female. Prevalence levels for MSM, commercial sex workers and pregnant women were 9.0%, 0.8%, and 0.2%, respectively. The expansion pathway resulting from this analysis showed, blood safety, peer education of sex workers, peer education of youth in school and voluntary counselling and testing to be the most cost effective order.

## Discussion

The primary aim of this analysis was to determine how an intervention response to HIV could be most cost effectively implemented in Peru. This analysis provides estimates of historical and future levels of HIV based on the best available data and using methods currently recommended by UNAIDS in producing country estimates. Using these baseline prevalence estimates, an evaluation of the cost effectiveness of a series of separate interventions aimed at reducing the levels of HIV transmission for the period 2008 - 2013 has been carried out.

Estimates for the number of people living with HIV in Peru vary widely depending on the source, and range from 20,000 to 144,000 [23]. Levels predicted by this work for 2007 were 62,450 which are between these limits and in the lower range of those predicted by UNAIDS (57,000 to 97,000 people living with HIV/AIDS for 2007)[24] for the same year.

In 1993 the first global comparison of a large number of interventions against a range of diseases determined that interventions costing less than US$1000 per DALY averted were cost-effective[25]. However, a more up to date set of criteria suggested by the Commission on Macroeconomics and Health states that interventions costing less than three times GDP per capita to avert each DALY are cost-effective[26]. Using the second of these two definitions of cost effectiveness, and considering Peru's GDP per capita was US$7,600 in year 2007[27], all of the interventions included in this study would be regarded as cost-effective.

The inclusion of HAART within this analysis may be considered controversial. After years of campaigning for access to treatment we are not intending to undermine HIV positive Peruvians their fundamental right to treatment. However, the finding that HAART is one of the more costly interventions will come as no surprise and is consistent with other studies using similar methodologies[13]. Whilst HAART is one of the most expensive options, it remains cost effective by the definitions outlined above and without any doubt should be included in any package against HIV in Peru. Importantly, our estimates of average cost effectiveness ratios (between $US 1995 for a basic programme to $US 2403 for a comprehensive package $/DALY averted) are in a similar range to those presented in another study that estimated cost effectiveness for antiretroviral therapy for Africa in 2002 as between $US1100 and $US1800[28]. Another study looking at the regions of sub-Saharan Africa and South East Asia found that antiretroviral therapy in these areas was calculated to cost between $Int 542 and $Int 2010 - $Int being a hypothetical currency that have the same purchasing power that the $US has in the United States at any given point[13].

As with any exercise in cost-effectiveness estimation, this analysis is not free from limitations which warrant caution when considering the implications of the results presented. Deciding what interventions to include was determined by three factors: whether the intervention was included in the GOALS model; whether sufficient information was available for its inclusion in the model[19]; and whether an intervention was deemed locally appropriate. The set of interventions chosen as a result of this work may not therefore represent the best case scenario and more appropriate options may exist. It is important to note that of all the studies used in determining the impact of interventions within the GOALS model, only very few of these were actually carried out in Latin America[18,29-37]. Although reproducibility in Latin America should be possible, their impact may vary in comparison to the initially reported studies, which would have an effect on their cost effectiveness when applied in Peru.

The higher risk groups which the interventions in this model are targeted at are presented in this paper as discrete cohorts. However, as many papers in Peru have suggested, this categorisation of people based on certain behaviours or sexual preferences not only excludes certain groups at higher risk, but includes others inappropriately [38-41]. The GOALS model, as it currently stands, does not allow for full mixing between the at risk groups over the analysis period which would differ from reality, as for example, some high risk will move to the low risk group over the time course of the analysis. This lack of sub-categorisation, mixing over time of the groups within the analysis over time, and the associated limitations is acknowledged, however, carrying out the analysis in this way allows a certain amount of comparability across studies by replicating methods and techniques applied elsewhere [5,6,10,13,19,42].

It is suggested that due to levels of uncertainty in the assumptions used in the model, interpretation of results should be based on broad categories of cost effectiveness rather than the absolute levels of infections preventable per intervention. The rankings suggested by this analysis are meant as a guide only and the exact order should not be taken as an absolute, as demonstrated by the sensitivity analysis. The values presented in table 5 should not be compared to current epidemiological estimates as they do not take into account existing interventional impacts on condom usage, treatment of STIs and reduction in high risk behaviour, which were set to zero. As a result, data must be interpreted with caution by policy makers as it does not include interventions which are currently occurring. The necessary information required to make such an assessment of interventions compared to current efforts levels is unfortunately lacking in many areas at present.

Apart from the uncertainties within the analysis, there are other legal and social factors to consider when weighing up the results of this cost effectiveness analysis such as the increasing perception of certain services as rights and social legitimacy. Such factors are even more pertinent for the MSM and FSW interventions where the stigma associated with membership of these groups leads to the avoidance of people assuming the identity of these at risk groups. Although the uncertainty analysis in the study goes some way to addressing infectivity and the clinical stage of HIV/AIDS, practical issues such as these which impact on clinical management of patients, were ignored by this analysis.

The ability of HAART to impact on sexual behaviour and transmissibility was not included in this analysis as this facility is not currently included in the interventional impacts in the GOALS model. Consequently this analysis may underestimate the actual cost effectiveness of this intervention. Precise and reliable estimates regarding the behavioural and biological inputs required for the model were limited and many figures used in the analysis were based on studies of small numbers of subjects. In some areas in particular, such as number of coital acts per year and number of partners per year for some of the risk groups, no data existed resulting in assumptions being made regarding this data. As called for in another article [4] this data should be collected as a matter of priority in order that more accurate analysis is carried out in the near future.

Unit costs calculated by the analysis were substantially influenced by personnel and peer educator costs. Final results are therefore sensitive to assumptions about these values. Values for the unit costs per person reached varied from $US 8 per youth reached up to $US 3792 for a comprehensive HAART programme. These HAART values appeared reasonable when compared with other such calculations (estimated values between $US395 and $US 5400 for various countries in Latin America)[5,43,44] and on the whole represented good value for money.

The expansion pathway, calculated by adding interventions to each other in order of their cost effectiveness, gives an idea of the levels of synergy that occur by having multiple intervention strategies and the costs associated with such packages of interventions. As with another study[13], the expansion pathway follows an exponential trajectory as the more expensive interventions are added to the overall strategy.

Peer education of sex workers, mass media, treatment of STIs and voluntary counselling and testing were found to be the four most cost effective interventions in a similar analysis based on South East Asia[13] - blood safety was not included in this analysis. The results of this study suggest that peer education of sex workers, blood safety, peer education of youth in school and voluntary counselling and testing would all substantially reduce the burden of HIV/AIDS in Peru and are economically attractive when using the international accepted definitions based on cost per DALY averted. Although the epidemic of HIV in Peru presents a concentrated high-risk group pattern, with MSM presenting the highest concentration of prevalence, blood safety was shown to be a very cost effective measure because of its broad population based effect. It is important to note that since coverage of blood screening is already nearly 100%, the conclusion we suggest is drawn from this result is that the model supports that blood screening should continue because it is so cost-effective, not that more infections could be averted by better blood screening.

STIs within the model are broadly split into non-ulcerative and ulcerative. This simplification assumes that HSV2 (along with syphilis and chancroid with which it is classified) is curable and has a higher rate of transmission than it does in reality, which is noted as a limitation of this particular model by the authors. We also note the current growing consensus that treatment of STIs, especially in generalized epidemics, appears to have a minimal population level effect, and the growing evidence to support male circumcision which is not currently included within the GOALS model[45].

Interventions targeted at MSM consistently performed well in terms of total infections averted, ranking in the top three, however, it did not perform well in terms of cost effectiveness. Much of this lack of performance can be explained by the inability of the model to compute specific sub-populations of MSM - classifying them as a single homogeneous at risk group ignores the reality of varying behavioural and risk characteristics[39,40,46-48]. If interventions were targeted more specifically at these groups then numbers of participants included would be smaller and the interventions would likely to improve significantly in the cost effectiveness rankings. Unusually for a concentrated epidemic, youth education in school has a large impact on the number of infections averted. This appears to be mainly due to sensitivity of the model to interventions on medium risk groups, which although representing a smaller prevalence, represent a large number of sexual acts performed.

## Conclusion

This analysis has determined how an intervention response to HIV could be most cost effectively implemented in Peru. HIV prevalence levels over time for various at risks groups were estimated; country-specific unit costs for a set of prevention and treatment interventions were calculated and a potential cost-effective expansion pathway is presented. The results of this work add evidence-based clarity as to which interventions warrant greatest consideration when planning an intervention response to HIV in Peru. Cost effectiveness analysis provides a necessary element of transparency when facing choices about priority setting with limited resources, but is equally important in an era of unprecedented resources for HIV in Peru and many other countries, and should be considered together with criteria regarding social inclusion and the universal right to health. Likewise, new evidence regarding effectiveness of specific interventions is fundamental, as is the design of new structural interventions to alter their social origin (such as those that create vulnerability) rather than its effects.

## Competing interests

The authors declare that they have no competing interests.

## Authors' contributions

RWA reviewed available data and literature, analysed data, and wrote the manuscript. DI and CFC provided data and editorial comments on the manuscript. JJM provided data, guidance on the analysis, and editorial comments on the manuscript. All authors read and approved the final manuscript.

## Pre-publication history

The pre-publication history for this paper can be accessed here:



## Supplementary Material

Additional file 1 **Supplementary material for article: Determining a cost effective intervention response to HIV/AIDS in Peru**. This supplementary material contains additional detail of the data used in the analysis and not presented in the main article. Click here for file
