# Supplementary material for: Determining a cost effective intervention response to HIV/AIDS in Peru
Source: BMC Public Health. 2009 Sep 18;9:352. doi: 10.1186/1471-2458-9-352 (PMC2761404; doi:10.1186/1471-2458-9-352)
Supplement: Additional file 1 — Supplementary material for article: Determining a cost effective intervention response to HIV/AIDS in Peru. This supplementary material contains additional detail of the data used in the analysis and not presented in the main article. [file 1471-2458-9-352-S1.doc]

**Supplementary material for article: Determining a cost effective intervention response to HIV/AIDS in Peru**

Robert W. Aldridge, David Iglesias, Carlos F. Cáceres, J. Jaime Miranda

**Table 1 Unit costs associated with intervention targeted at MSM (Costs in $US)**.

| **MSM - peer workers** | |  |  |
| --- | --- | --- | --- |
|  | **Detail** | **Assumption** | **Reference** |
|  | Peer educator to MSM worker ratio | 20 | 1,2 |
|  | Peer educator remuneration per day | 15 | 2 |
|  | Peer educator days to work per week | 2 | 1,2 |
|  |  |  |  |
| **Peer worker training** | |  |  |
|  | **Detail** | **Assumption** | **Reference** |
|  | Number of trainers required to organise training course for peer workers | 2 | 1,2 |
|  | Number of days to complete one course | 2 | 1,2 |
|  | Number of peer workers in one group training session | 20 | 1,2 |
|  | Daily allowance to attend training - e.g. travel and accommodation | 15 | 2 |
|  | Trainer fees per day | 30 | 2 |
|  | Materials cost per participant per course | 5 | 2 |
|  | Food and refreshment costs per participant per day | 10 | 2 |
|  |  |  |  |
| **Outreach workers** | |  |  |
|  | **Detail** | **Assumption** | **Reference** |
|  | Number of outreach workers per peer educator | 17 | 1,2 |
|  | Monthly salary of outreach worker | 300 | 2 |
|  |  |  |  |
| **Condoms and Lubricants** | |  |  |
|  | **Detail** | **Assumption** | **Reference** |
|  | Cost per male condom | 0.02 | 2 |
|  | Average number of partners per MSM per day | 1 | 3 |
|  | Average number of days of sexual activity per year | 60 | 3 |
|  | % of condoms used consistently | 70 | 3 |
|  | Buffer stocks of condoms required | 30 | 1,2 |
|  |  |  |  |
| **STI services** | |  |  |
|  | **Detail** | **Assumption** | **Reference** |
|  | Number of doctors per STI unit | 1 | 1,2 |
|  | Number of nurses per STI unit | 1 | 1,2 |
|  | Monthly doctor salary | 1,200 | 2 |
|  | Monthly nurse salary | 800 | 2 |
|  | Monthly assistant salary | 600 | 2 |
|  | Time/patient (minutes) for examination and treatment | 30 | 1,2 |
|  | Average cost of drugs per treatment per episode | 3.67 | 4 |
|  | Assumed STI prev. % | 9.00 | 3 |
|  | Assumed STI/MSM/year | 9.00 | 3 |

Joint United Nations Programme on HIV/AIDS (UNAIDS), Asian Development Bank (ADB): Costing Guidelines for HIV/AIDS Intervention Strategies. 1st edition. Geneva: UNAIDS, ADB; 2004.

^2^ Expert opinion based upon either published Peruvian data, field experience of local experts working at local NGOs that the authors of this paper had discussions with, or from direct programme experience of the Peruvian-based authors.

^3^ Output from the GOALS model run as part of the best fit analysis.

^4^ Adams EJ, Garcia PJ, Garnett GP, Edmunds WJ, Holmes KK: The cost-effectiveness of syndromic management in pharmacies in Lima, Peru. Sexually transmitted diseases 2003, 30(5):379-387.

**Table 2 Unit costs associated with intervention targeted at FSW (Costs in $US).**

| **FSW - peer workers** | |  |  |
| --- | --- | --- | --- |
|  | **Detail** | **Assumption** | **Reference** |
|  | Peer educator to FSW worker ratio | 20 | 1,2 |
|  | Peer educator remuneration per day | 15 | 2 |
|  | Peer educator days to work per week | 2 | 1,2 |
|  |  |  |  |
| **Peer worker training** | |  |  |
|  | **Detail** | **Assumption** | **Reference** |
|  | Number of trainers required to organise training course for peer workers | 1 | 1,2 |
|  | number of days to complete one course | 2 | 1,2 |
|  | Number of peer workers in one group training session | 20 | 1,2 |
|  | Daily allowance to attend training - e.g. travel and accommodation | 15 | 2 |
|  | Trainer fees per day | 30 | 2 |
|  | Materials cost per participant per course | 5 | 2 |
|  | Food and refreshment costs per participant per day | 10 | 2 |
|  |  |  |  |
| **Outreach workers** | |  |  |
|  | **Detail** | **Assumption** | **Reference** |
|  | Number of outreach workers per peer educator | 17 | 1,2 |
|  | Monthly salary of outreach worker | 300 | 2 |
|  |  |  |  |
| **Condoms and Lubricants** | |  |  |
|  | **Detail** | **Assumption** | **Reference** |
|  | Cost per male condom | 0.02 | 2 |
|  | Average number of clients per FSW per day | 4 | 3 |
|  | Average number of days of sex work activity per year | 250 | 3 |
|  | Percentage of condoms used consistently | 84 | 3 |
|  | Buffer stocks of condoms required (%) | 30 | 1,2 |
|  |  |  |  |
| **STI services** | |  |  |
|  | **Detail** | **Assumption** | **Reference** |
|  | Number of doctors per STI unit | 1 | 1,2 |
|  | Number of nurses per STI unit | 1 | 1,2 |
|  | Monthly doctor salary | 1,200 | 2 |
|  | Monthly nurse salary | 800 | 2 |
|  | Monthly assistant salary | 600 | 2 |
|  | Time/patient (minutes) for examination and treatment | 30.00 | 1,2 |
|  | Average cost of drugs per treatment per episode | 3.67 | 4 |
|  | Assumed STI prev. % | 5.30 | 3 |
|  | Assumed STI/SW/year | 5.30 | 3 |

Joint United Nations Programme on HIV/AIDS (UNAIDS), Asian Development Bank (ADB): Costing Guidelines for HIV/AIDS Intervention Strategies. 1st edition. Geneva: UNAIDS, ADB; 2004.

^2^ Expert opinion based upon either published Peruvian data, field experience of local experts working at local NGOs that the authors of this paper had discussions with, or from direct programme experience of the Peruvian-based authors.

^3^ Output from the GOALS model run as part of the best fit analysis.

^4^ Adams EJ, Garcia PJ, Garnett GP, Edmunds WJ, Holmes KK: The cost-effectiveness of syndromic management in pharmacies in Lima, Peru. Sexually transmitted diseases 2003, 30(5):379-387.

**Table 3 Unit costs associated with intervention targeted at VCT (Costs in $US).**

| **Training of counsellors** | |  |  |
| --- | --- | --- | --- |
|  |  |  |  |
|  | **Detail** | **Assumption** | **Reference** |
|  | Number of trainers required to organise training course for peer workers | 2 | 1,2 |
|  | Number of days to complete one course | 2 | 1,2 |
|  | Number of peer workers in one group training session | 20 | 1,2 |
|  | Daily allowance to attend training - e.g. travel and accommodation | 15 | 2 |
|  | Trainer fees per day | 30 | 2 |
|  | Materials cost per participant per course | 5 | 2 |
|  | Food and refreshment costs per participant per day | 10 | 2 |
|  |  |  |  |
|  |  |  |  |
|  |  |  |  |
| **Voluntary counselling and testing - activities** | |  |  |
|  |  |  |  |
|  | **Detail** | **Assumption** | **Reference** |
|  | Counsellors per VCT unit | 10 | 1,2 |
|  | Monthly salary per counsellor | 450 | 2 |
|  | Counselling Capacity used % of time counsellors spend counselling | 75 | 1,2 |
|  |  |  |  |
| **Commodities and services** | |  |  |
|  |  |  |  |
|  | **Detail** | **Assumption** | **Reference** |
|  | Assumed HIV prevalence | 0.60 | 3 |
|  | Single test HIV | 1.20 | 2 |
|  | Confirmation test HIV | 8.92 | 2 |
|  | Cost per male condom | 0.02 | 2 |

Joint United Nations Programme on HIV/AIDS (UNAIDS), Asian Development Bank (ADB): Costing Guidelines for HIV/AIDS Intervention Strategies. 1st edition. Geneva: UNAIDS, ADB; 2004.

^2^ Expert opinion based upon either published Peruvian data, field experience of local experts working at local NGOs that the authors of this paper had discussions with, or from direct programme experience of the Peruvian-based authors.

^3^ Output from the GOALS model run as part of the best fit analysis.

**Table 4. Unit costs associated with intervention targeted at PMTCT (Costs in $US).**

| **Training of counsellors** | |  |  |
| --- | --- | --- | --- |
|  |  |  |  |
|  | **Detail** | **Assumption** | **Reference** |
|  | Number of trainers required to organise training course for peer workers | 2 | 1,2 |
|  | Number of days to complete one course | 2 | 1,2 |
|  | Number of peer workers in one group training session | 20 | 1,2 |
|  | Daily allowance to attend training - e.g. travel and accommodation | 15 | 2 |
|  | Trainer fees per day | 30 | 2 |
|  | Materials per participant per course | 5 | 2 |
|  | Food and refreshment costs per participant per day | 10 | 2 |
|  |  |  |  |
|  |  |  |  |
| **Prevention of mother to child transmission** | |  |  |
|  |  |  |  |
|  | **Detail** | **Assumption** | **Reference** |
|  | Women requiring counselling per year per site | 100 | 1,2 |
|  | Staff salary per month | 800 | 2 |
|  | Staff hours per treatment | 1 | 1,2 |
|  | Cost of drugs administered per mother and child pair if HIV +ve | 22 | 2 |
|  | Number of tests required per mother and child pair | 2 | 1,2 |
|  | Cost of one test kit | 15 | 2 |

Joint United Nations Programme on HIV/AIDS (UNAIDS), Asian Development Bank (ADB): Costing Guidelines for HIV/AIDS Intervention Strategies. 1st edition. Geneva: UNAIDS, ADB; 2004.

^2^ Expert opinion based upon either published Peruvian data, field experience of local experts working at local NGOs that the authors of this paper had discussions with, or from direct programme experience of the Peruvian-based authors.

**Table 5. Unit costs associated with intervention targeted at Youth – in school (Costs in $US).**

| **Orientation costs** | |  |  |
| --- | --- | --- | --- |
|  |  |  |  |
|  | **Detail** | **Assumption** | **Reference** |
|  | Number of people for orientation | 500 | 1,2 |
|  | Average number of people per session | 50 | 1,2 |
|  | Remuneration per participant per orientation | 2 | 1,2 |
|  |  |  |  |
| **Training of counsellors** | |  |  |
|  |  |  |  |
|  | **Detail** | **Assumption** | **Reference** |
|  | Number of trainers required to organise training course for peer workers | 1 | 1,2 |
|  | Number of days to complete one course | 2 | 1,2 |
|  | Number of peer workers in one group training session | 20 | 1,2 |
|  | Daily allowance to attend training - e.g. travel and accommodation | 15 | 2 |
|  | Trainer fees per day | 30 | 2 |
|  | Materials per participant per course | 5 | 2 |
|  | Food and refreshment costs per participant per day | 10 | 2 |
|  |  |  |  |
| **supervision** | |  |  |
|  |  |  |  |
|  | **Detail** | **Assumption** | **Reference** |
|  | Number of supervisors required | 1 | 1,2 |
|  | Supervisor salary per month | 450 | 2 |
|  | Number of days per location per year | 2 | 1,2 |
|  | Number of visits per location per year | 2 | 1,2 |
|  | Monthly salary of outreach worker | 300 | 2 |

Joint United Nations Programme on HIV/AIDS (UNAIDS), Asian Development Bank (ADB): Costing Guidelines for HIV/AIDS Intervention Strategies. 1st edition. Geneva: UNAIDS, ADB; 2004.

^2^ Expert opinion based upon either published Peruvian data, field experience of local experts working at local NGOs that the authors of this paper had discussions with, or from direct programme experience of the Peruvian-based authors.

**Table 6. Unit costs associated with intervention targeted at Mass media (Costs in $US).**

| **Mass media costs** | |  |  |
| --- | --- | --- | --- |
|  |  |  |  |
|  | **Detail** | **Assumption** | **Reference** |
|  | Information leaflets A4 size distributed to 7% of population annually | 1,015,000 | 1,2 |
|  | 13 emissions of each 5 minutes on national TV annually | 25,773 | 1,2 |
|  | 45 emissions of each 2.5 minutes on 5% of all radio stations in region annually | 558,262 | 1,2 |

Joint United Nations Programme on HIV/AIDS (UNAIDS), Asian Development Bank (ADB): Costing Guidelines for HIV/AIDS Intervention Strategies. 1st edition. Geneva: UNAIDS, ADB; 2004.

^2^ Expert opinion based upon either published Peruvian data, field experience of local experts working at local NGOs that the authors of this paper had discussions with, or from direct programme experience of the Peruvian-based authors.

**Table 7 Unit costs associated with intervention targeted at ARV**

| **Package** | **Component** | **$US** |
| --- | --- | --- |
|  |  |  |
| Basic |  |  |
|  | Antiretrovirals | 2645 |
|  | Monitoring | 734 |
|  | **Total** | **3379** |
|  |  |  |
| Intermediate |  |  |
|  | Antiretrovirals | 2532 |
|  | Monitoring | 703 |
|  | Others lab tests | 299 |
|  | Imaging | 29 |
|  | Admin | 225 |
|  | **Total** | **3788** |
|  |  |  |
| Comprehensive |  |  |
|  | Antiretrovirals | 2530 |
|  | Monitoring | 702 |
|  | Others lab tests | 299 |
|  | Imaging | 29 |
|  | Admin | 225 |
|  | Opportunistic infection treatmeny | 7 |
|  | **Total** | **3792** |

Figures presented above are calculated from the analysis of three alternative treatment regimes in a report addressing the implementation of an antiretroviral programme for Peru[[1]](#footnote-2). Unit costs are per person per year.

**Table 8. Matrices used in the GOALS model detailing impact of exposure to a prevention intervention on: the reduction in non-use of condoms, reduction in non treatment of STIs, reduction in number of partners and rise in age at first sex. Average impact (medium), lower quartile (Low) and upper quartile (High) details are shown[[2]](#footnote-3)**.

**Table 9 Assumptions made about HIV, STI and mother to child transmission probabilities**

**Sexually transmitted infections**

Duration of infectiousness of untreated STD (months) 1.2

Duration of infectiousness of treated STD (months) 0.5

Ratio of STI incidence to prevalence 1.6

Condom efficacy 80%

| **STD transmission probabilities per contact** |  |
| --- | --- |
| GUD | 0.2 |
| Non-GUD | 0.2 |
| **HIV transmission per contact** |  |
| Female to male, no STI, no circumcision, chronic phase of infection | 0.0018 |
| Multipliers on HIV transmission per contact: |  |
| Male to female | 1 |
| Presence of GUD | 8 |
| Presence of non-GUD STI | 4 |
| Acute stage of infection | 8 |
| End stage of infection | 4 |
| Circumcised | 0.42 |
| Male to male, no STI, no circumcision, chronic phase of infection | 0.01 |
|  |  |
| Duration of acute phase of infection (years) | 0.1 |
| Duration of end phase of infection (years) | 2.0 |
| Duration of infection (time from infection until death without ART) | 9.0 |

| **Mother-to-child transmission** |  |
| --- | --- |
| Probability of transmission with no treatment | 0.3 |
| Probability of transmission with treatment and BF | 0.23 |
| Probability of transmission with Tx and BF replacement | 0.13 |

1. Ministerio de Salud del Peru, Universidad Peruana Cayetano Heredia: Recommendations for the Implementation of an HIV/AIDS Antiretroviral Therapy Program in Peru. First edition.: Ministerio de Salud del Peru; 2004. [↑](#footnote-ref-2)
2. Stover J BL, Cooper-Arnold K.: Goals Model: For estimating the effects of resource allocation decisions on the achievement of the goals of the HIV/AIDS strategic plan. Glastonbury: The Futures Group International; 2003. [↑](#footnote-ref-3)
